# Cur@ZIF-8@BA nanomaterials with pH-responsive and photodynamic therapy properties promotes antimicrobial activity

**DOI:** 10.3389/fchem.2024.1417715

**Published:** 2024-06-24

**Authors:** Xiujuan Shang, Hongdong Wang, Yongbo Yu, Jin Gu, Jian Zeng, Sinan Hou

**Affiliations:** ^1^ Department of Laboratory Medicine, Lianyungang Affiliated Hospital of Nanjing University of Chinese Medicine, Lianyungang, Jiangsu, China; ^2^ Lianyungang Maternal and Child Health Hospital, Lianyungang, Jiangsu, China; ^3^ Kangda College of Nanjing Medical University, Lianyungang, Jiangsu, China

**Keywords:** antibacterial photodynamic therapy, curcumin, ZIF-8, boric acid, nanomedicine

## Abstract

Antimicrobial photodynamic therapy (aPDT) has emerged as a highly promising strategy for non-antibiotic treatment of infections due to its unique advantages in efficient bactericidal action and reduction of drug resistance. The natural photosensitizing properties of curcumin (Cur) are widely acknowledged; however, its limited bioavailability has impeded its practical application. In this study, we developed a nanomaterial called Cur@ZIF-8@BA by encapsulating Cur within ZIF-8 and modifying the surface with boric acid (BA). The Cur@ZIF-8@BA exhibits pH-responsive properties and enhances bacterial binding, thereby effectively promoting photodynamic therapy. Moreover, its antibacterial activity against *E. coli*, *Staphylococcus aureus* and *A. baumannii* is significantly increased in the presence of light compared to a dark environment. The mechanism behind this may be that BA increases the affinity of Cur@ZIF-8@BA towards bacteria, and making released Zn^2+^ and BA from the nanomaterial increase bacterial cell membrane permeability. This facilitates efficient delivery of Cur into bacterial cells, resulting in generation of abundant reactive oxygen species (ROS) and subsequent bactericidal activity. In conclusion, our prepared Cur@ZIF-8@BA holds great promise as a photodynamically mediated antimicrobial strategy.

## 1 Introduction

Bacterial infections have emerged as a paramount global public health challenge, exerting an immense burden on national healthcare systems (Ramos et al., 2019). Particularly in the face of multidrug-resistant bacteria, patients frequently encounter limited access to efficacious treatment modalities, ultimately succumbing to their condition (Mizobuchi et al., 2019). Projections indicate that if left unaddressed, over 10 million individuals worldwide will perish annually from multidrug-resistant bacterial infections by 2050 (Davis et al., 2022). Consequently, the clinical treatment of multi-drug resistant bacterial infections necessitates the development of a non-antibiotic based alternative therapy as an urgent requirement.

Antimicrobial photodynamic therapy (aPDT) has emerged as a highly promising strategy for non-antibiotic treatment of infections due to its unique advantages in efficient bactericidal action and reduction of drug resistance (Piksa et al., 2023). In aPDT therapy, photosensitizers can rapidly generate a substantial amount of reactive oxygen species (ROS) under optimal light conditions, leading to the disruption of bacterial cell membranes and DNA, ultimately resulting in bacterial death (Awad et al., 2022). Therefore, the antibacterial efficacy of aPDT primarily relies on the properties of its own photosensitizer. However, many photosensitizers still face challenges such as limited water solubility, significant self-quenching effects, and poor stability that require immediate attention (Zhang et al., 2022). As widely acknowledged, curcumin (Cur) is a polyphenolic compound derived from turmeric that has garnered significant attention from researchers due to its diverse pharmacological activities, encompassing anti-inflammatory, antioxidant, anti-tumor, and antibacterial properties (Lin et al., 2022). Notably, Cur also exhibits remarkable potential as a natural photosensitizer, which may contribute to its antibacterial efficacy (Wang et al., 2022). However, the clinical application of Cur in the field of antibacterials remains severely limited primarily due to its extremely low solubility, poor photothermal stability, and inadequate bioavailability (Duan et al., 2022). An effective approach to overcome these challenges involves encapsulating Cur within suitable drug delivery systems capable of achieving targeted controlled release for enhanced water solubility, stability, and bioavailability (Ben Yehuda Greenwald et al., 2017).

The zeolitic imidazolate framework-8 (ZIF-8), a prominent star in the field of metal-organic frameworks (MOFs), possesses numerous advantages including high porosity, large specific surface area, well-defined crystalline pores, and tunable pore sizes, and is widely used in the field of medicine (Zhang et al., 2022). Research has demonstrated that ZIF-8 exhibits pH-sensitive release properties within slightly acidic environments associated with bacterial infections, enabling targeted delivery (Zhao et al., 2022). Moreover, the released Zn^2+^ ions from ZIF-8 decomposition can effectively eradicate bacteria by disrupting their cell membrane functionality, penetrating into the intracellular space to interfere with enzyme activity and metabolism (Wu et al., 2019). A few studies have also reported that reactive oxygen species (ROS) can be generated following the action of ZIF-8 to achieve a bactericidal effect (Shen et al., 2022). Therefore, ZIF-8 serves as an optimal carrier for drug delivery and antibacterial purposes, enabling the encapsulation of Cur and facilitating its controlled release within the microenvironment of bacterial infection. This approach can enhance the stability and bioavailability of Cur while achieving a synergistic antibacterial effect.

It should be noted that in the face of the intricate bacterial infection environment, current non-antibiotic antimicrobial strategies typically necessitate multidirectional collaborative approaches to enhance antibacterial efficacy (Zhang et al., 2023). The release properties of ZIF-8 are pH-dependent in the weak acid environment of bacterial infection. However, it lacks direct targeting towards bacteria themselves, necessitating further optimization to enhance its performance. Studies have demonstrated that apart from its well-established antibacterial activity, boric acid (BA) possesses a crucial ability whereby its surface groups can covalently bind with abundant glycoproteins and carbohydrates on the bacterial cell wall, thereby significantly enhancing the affinity between BA-modified antibacterial agents and bacteria and consequently improving bactericidal efficiency to a great extent (Chen et al., 2022). Moreover, BA is also recognized for its low *in vivo* toxicity, high stability, and ease of handling (Halbus et al., 2019). Clearly, incorporating BA modification onto ZIF-8 holds promise for augmenting ZIF-8’s ability to specifically target bacteria and overall enhance bactericidal efficiency.

**SCHEME 1:**
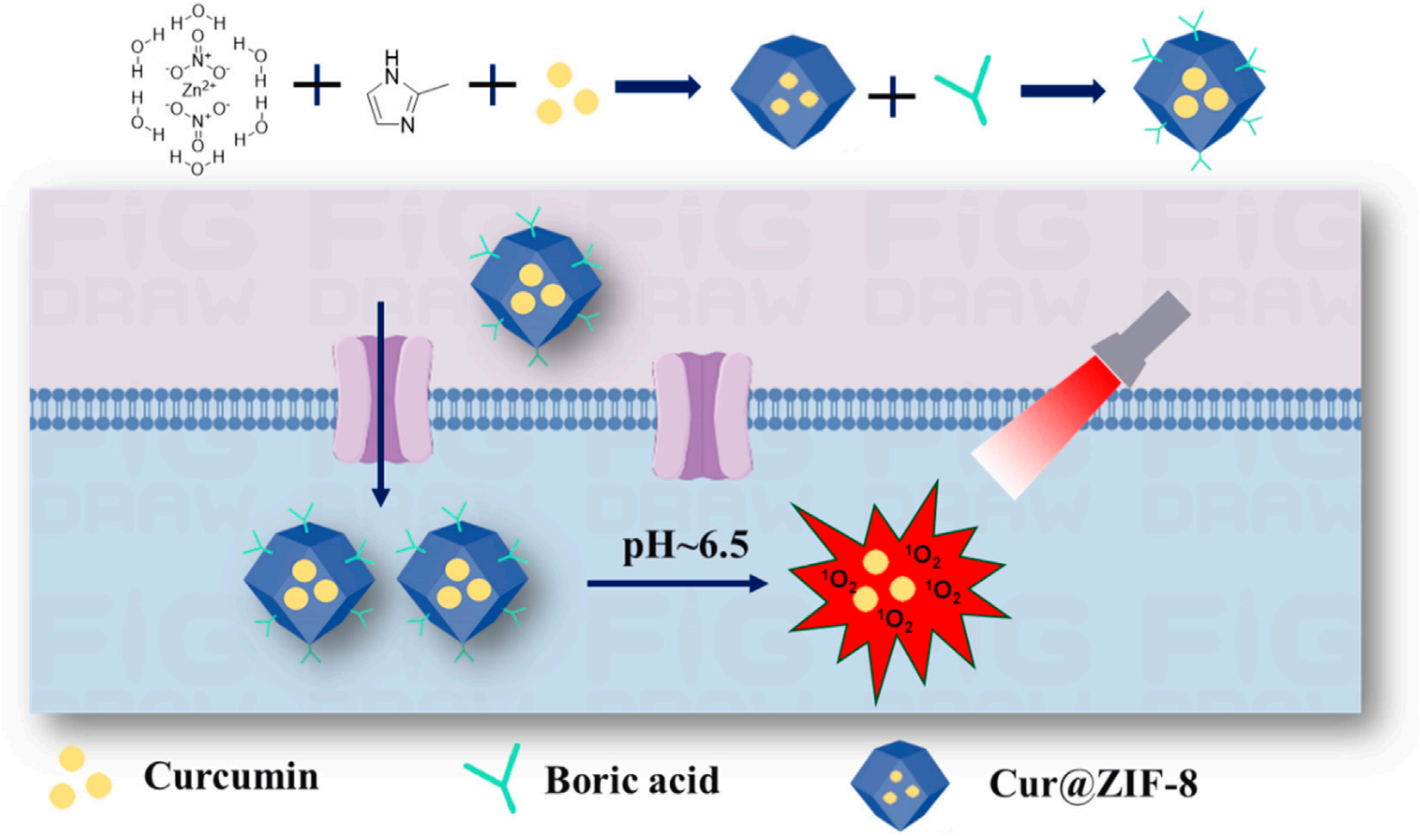
The schematic diagram illustrating the synthesis process and antibacterial mechanism of Cur@ZIF-8@BA.

Herein, we have developed a BA-modified nano-antibacterial material called Cur@ZIF-8@BA (Scheme 1). This material encapsulates the natural photosensitizer Cur within ZIF-8 to enhance its water solubility, improve light stability, and increase bioavailability. By utilizing the pH-responsive property of ZIF-8, it can degrade and release both Zn^2+^ and Cur in the weakly acidic environment of bacterial infections, thereby exerting a synergistic antibacterial effect. Lastly, the modification of Cur@ZIF-8 with BA enhances its affinity towards bacteria, leading to a significant enhancement in overall bactericidal efficiency. In summary, this study proposes a promising non-antibiotic strategy to address the urgent issue of bacterial infections.

## 2 Materials and methods

### 2.1 Materials

*Staphylococcus aureus* (strain ATCC 25923), *E. coli* (strain ATCC 25922), and A. baumannii (strain ATCC 19606) were acquired from Shanghai Fuxiang Biotechnology Co., Ltd. NIH 3T3 (CL-0171) cells was procured from Wuhan Pricella Biotechnology Co., Ltd., Wuhan, China. The DCFH-DA probe (HY-D0940) and the ABDA probe (MX4822-50MG) were acquired from Shanghai MedChemExpress and Shanghai MAOKANG Biotechnology, respectively. Others general qualitative analysis-grade chemicals were purchased from Aladdin (China) without the need for additional purification.

### 2.2 Preparation of Cur@ZIF-8@BA

ZIF-8 was synthesized following the reported method (Li et al., 2023). The synthesis of Cur@ZIF-8 was conducted as per the following procedure: Firstly, 150 mg of zinc nitrate hexahydrate was dissolved in 5 mL of deionized water. Next, 2-methylimidazole (330 mg) and Cur (5 mg) were dissolved in methanol (10 mL). The two solutions were mixed and stirred for 5 min, followed by centrifugation at a speed of 10,000 rpm/min for 15 min. Subsequently, thorough washing with methanol was performed to eliminate any unreacted components. The resulting milky Cur@ZIF-8 product was dried under vacuum conditions and stored for future use.

Dissolve 100 mg of Cur@ZIF-8 and 20 mg of (2,5-dicarboxyphenyl) BA in 10 mL of methanol, stir for 10 min, centrifuge at 10,000 rpm for 15 min, and thoroughly wash with methanol to remove any unreacted components. The resulting Cur@ZIF-8@BA composite material is then dried under vacuum and stored for future use.

### 2.3 Characterization of Cur@ZIF-8@BA

The morphologies, sizes and structure of ZIF-8, Cur@ZIF-8, and Cur@ZIF-8@BA were examined using a transmission electron microscope (JEM-1400 Flash, Japan) and X-ray diffractometer (D8 Advance, Bruker). The zeta units and particle size of these materials were measured using dynamic light scattering nanoparticles and a Zeta potential analyzer (ZS 920, Zimeng Technology Co., LTD., Shanghai). UV-vis spectra of the different samples were obtained using a UV-6100 (Mapada, Shanghai, China) type ultraviolet/visible spectrophotometer. Fluorescence spectra of the different samples were recorded with an F-7100 (Hitachi, Japan) fluorescence spectrophotometer.

### 2.4 Stability investigation of Cur@ZIF-8@BA

The 10 mg Cur@ZIF-8@BA were incubated in 1 mL of distilled water, PBS (pH 7.4), and DMEM medium for a duration of 24 h, respectively. Subsequently, the stability of the material in different media was assessed by measuring the particle size of Cur@ZIF-8@BA following incubation.

### 2.5 The drug loading content and drug loading efficiency of Cur and BA

The drug loading content (DLC) and drug loading efficiency (DLE) of Cur and BA in Cur@ZIF-8@BA were determined using a UV-visible spectrophotometer. 1 mg of Cur@ZIF-8@BA was mixed with 10 mL hydrochloric acid and 2 mL anhydrous ethanol, then treated with ultrasound for 10 min to extract Cur and BA from Cur@ZIF-8@BA. The absorbance of free Cur and BA at different concentrations was measured at 425 and 280 nm respectively to establish their standard curves. These standard curves were subsequently utilized to quantify the total amount of Cur and BA in the solution. The calculation formula for DLC and DLE is as follows:

$$D\text{LC }\left(\text{Cur}\right)\%=\frac{\text{Encapsulated}\;\text{Cur}\;\left(\text{mg}\right)}{T\text{otal}\;\text{composite}\;\text{nanoparticles}\;\left(\text{mg}\right)}\times 100\%$$

$$D\text{LE }\left(\text{Cur}\right)\%=\frac{\text{Encapsulated}\;\text{Cur}\;\left(\text{mg}\right)}{T\text{otal}\;\text{Cur}\;\text{input}\;\left(\text{mg}\right)}\times 100\%$$

$$D\text{LC }\left(\text{BA}\right)\%=\frac{\text{Encapsulated}\;\text{BA}\;\left(\text{mg}\right)}{T\text{otal}\;\text{composite}\;\text{nanoparticles}\;\left(\text{mg}\right)}\times 100\%$$

$$D\text{LE }\left(\text{BA}\right)\%=\frac{\text{Encapsulated}\;\text{BA}\;\left(\text{mg}\right)}{T\text{otal}\;\text{BA}\;\text{input}\;\left(\text{mg}\right)}\times 100\%$$

### 2.6 pH-responsive release of Cur@ZIF-8@BA

The pH responsiveness of the material was assessed by monitoring changes in particle size, release of Cur and Zn^2+^ in various pH media. Due to Cur’s insolubility in water and solubility in ethanol, a PBS buffer was mixed with an ethanol solution at a ratio of 4:1, and the pH was adjusted to 6.5 and 7.4 accordingly. Subsequently, 1 mg/mL ZIF-8, Cur@ZIF-8 and Cur@ZIF-8@BA were incubated in the aforementioned medium. The supernatant was collected at various time intervals (the volume extracted each time need to be replenished) for particle size determination as well as quantification of Cur and Zn^2+^. The standard curves for free Cur were established by measuring their absorbance at 425 nm, across different concentrations. Subsequently, the amount of Cur released from the material was quantified using these standard curves. Then the concentration of Zn^2+^ was determined by atomic absorption spectrometer (HITACHI Z-2000).

### 2.7 The ROS level examination

The level of reactive oxygen species (ROS) was assessed using the DCFH-DA probe. DCFH-DA, which lacks fluorescence signal, reacts with ROS to generate DCF with fluorescence absorption, and the fluorescence intensity at 525 nm is measured. The Cur@ZIF-8@BA (1 mg/mL) was incubated with 10 μM DCFH-DA in PBS, followed by exposure to light at an intensity of 100 mW/cm^2^. The change in fluorescence intensity at 525 nm was recorded over different durations of light exposure. The quantification of bacterial ROS levels was performed using a same protocol. Briefly, a diluted bacterial suspension was transferred to a sterile test tube and treated with BA, Cur, Cur@ZIF-8 and Cur@ZIF-8@BA (64 μg/mL) for 1 hour followed by exposure to 100 mW/cm^2^ light for an additional hour. Subsequently, the ROS levels were measured.

### 2.8 The detection of singlet oxygen levels

The singlet oxygen levels were quantified using the commercially available ABDA probe. The Cur@ZIF-8@BA (1 mg/mL) was incubated with 50 μM ABDA in PBS, followed by exposure to light at an intensity of 100 mW/cm^2^. The fluorescence intensity change at 378 nm was then measured at different time intervals.

### 2.9 Hemolytic tests

The red blood cells were obtained from fresh mouse blood through centrifugation (3,500 rpm/min, 5 min) and subsequently washed three times with a sodium thiobarbital solution to achieve a concentration of 5%. Different concentrations of Cur@ZIF-8@BA (128, 256, 512, 1,024, 2,048 μg/mL) were mixed with the aforementioned red blood cells and incubated at 37°C and 100 rpm for 1 h. Following this, the sample was subjected to centrifugation (3,500 rpm/min, 5 min). Finally, the absorbance of the supernatant at a wavelength of 545 nm was measured. PBS served as the negative control while deionized water served as the positive control. The hemolysis rate can be calculated using the following formula:

Hemolysis ratio (%) = [(OD_C_ − OD_P_)/(OD_W_-OD_P_)] ×100%. The abbreviations OD_C_, OD_P_, and OD_W_ represent Cur@ZIF-8@BA, PBS, and deionized water respectively.

### 2.10 The dark cytotoxicity of Cur@ZIF-8@BA

The *in vitro* safety of Cur@ZIF-8@BA was assessed using the Cell Counting Kit 8 (CCK-8). NIH 3T3 cells at logarithmic growth stage were seeded into 96-well plates at a density of 5 × 10^3^ cells/well. After cell attachment, varying concentrations of Cur@ZIF-8@BA (64,128, 256, 512, 1,024, 2,048 μg/mL) were added and incubated with the cells for 24 h. Subsequently, the medium was aspirated, and each well was treated with a solution containing 10% CCK-8. The OD value at a wavelength of 450 nm was measured after an additional incubation period of 2 h to determine the relative cell viability.

### 2.11 The determination of the minimum inhibitory concentration (MIC)

The MIC was determined using the two-tube dilution broth method (Baazeem et al., 2021). *S. aureus*, *E. coli* and *A. baumannii* were inoculated in LB medium and incubated at 37°C for 24 h. The bacterial diluent (1 × 10^8^ CFU/mL) was then transferred into sterile test tubes, followed by the addition of different concentrations of Cur@ZIF-8 and Cur@ZIF-8@BA for incubation for 1 h. Following this, the light group was exposed to a LED with an intensity of 100 mW/cm^2^ for 1 h before further incubation in the incubator for 22 h (Yu et al., 2017). The dark group did not undergo light treatment but was solely incubated in the incubator for a total duration of 24 h. Finally, the OD value at wavelength of 600 nm was measured for each group of solutions. The MIC is defined as the lowest concentration that inhibits the growth of 90% bacteria.

### 2.12 The test for antibacterial activity

Bacterial dilutions (1 × 10^8^ CFU/mL) of *S. aureus*, *E. coli* and *A. baumannii* were inoculated onto LB medium and incubated at 37°C for 24 h. Subsequently, Cur@ZIF-8 and Cur@ZIF-8@BA (64 μg/mL) were added, respectively. The subsequent light/dark processing is carried out in accordance with Method 2.11. The growth of bacteria was ultimately monitored and the colonies were quantified.

### 2.13 Monitoring the growth curve of bacteria

The 96-well plate was filled with 200 μL of MHB and 100 μL of bacterial diluter (1 × 10^6^ CFU/mL). Subsequently, Cur@ZIF-8 and Cur@ZIF-8@BA (64 μg/mL) were added. In the control group, an equal volume of PBS was added instead. The subsequent light/dark processing is carried out in accordance with Method 2.11. The plate was then incubated at 37°C. The initiation of drug therapy was recorded as time point zero (0), and subsequently, the optical density (OD) at a wavelength of 600 nm in the solution was measured at specific intervals to quantify the bacterial growth curve.

### 2.14 Bacterial biofilm clearance test

A dilute solution of 100 μL *S. aureus*, *E. coli* and *A. baumannii* (1 × 10^6^ CFU/mL) was added to 96-well plates for 24 h to facilitate biofilm formation. Subsequently, the bacterial solution was aspirated using a microsyringe and the biofilm was washed three times with PBS. The addition of Cur@ZIF-8 and Cur@ZIF-8@BA (64 μg/mL) is followed by subsequent light/dark processing, as outlined in Method 2.11. The treatment solution was then discarded before proceeding to the next step. The biofilm was ultimately subjected to treatment with a 95% ethanol solution, and subsequently, its OD value at 620 nm was measured to assess the material’s effectiveness in eradicating the biofilm.

### 2.15 Statistical analysis

The statistical analysis was conducted using one-way ANOVA, followed by LSD *post hoc* comparisons for data with equal variances, or Dunnett’s T3 for data with unequal variances. Two-tailed Student's *t*-tests were used to compare two groups. All analyses were performed using SPSS 21.0 software. A significance level of *p* < 0.05 was considered statistically significant. The results were presented as the mean ± standard error of the mean (SEM), and the experiment was replicated three times. Graphs were generated using GraphPad Prism 8.3.

## 3 Results and discussion

### 3.1 The characterization of Cur@ZIF-8@BA

**FIGURE 1:**
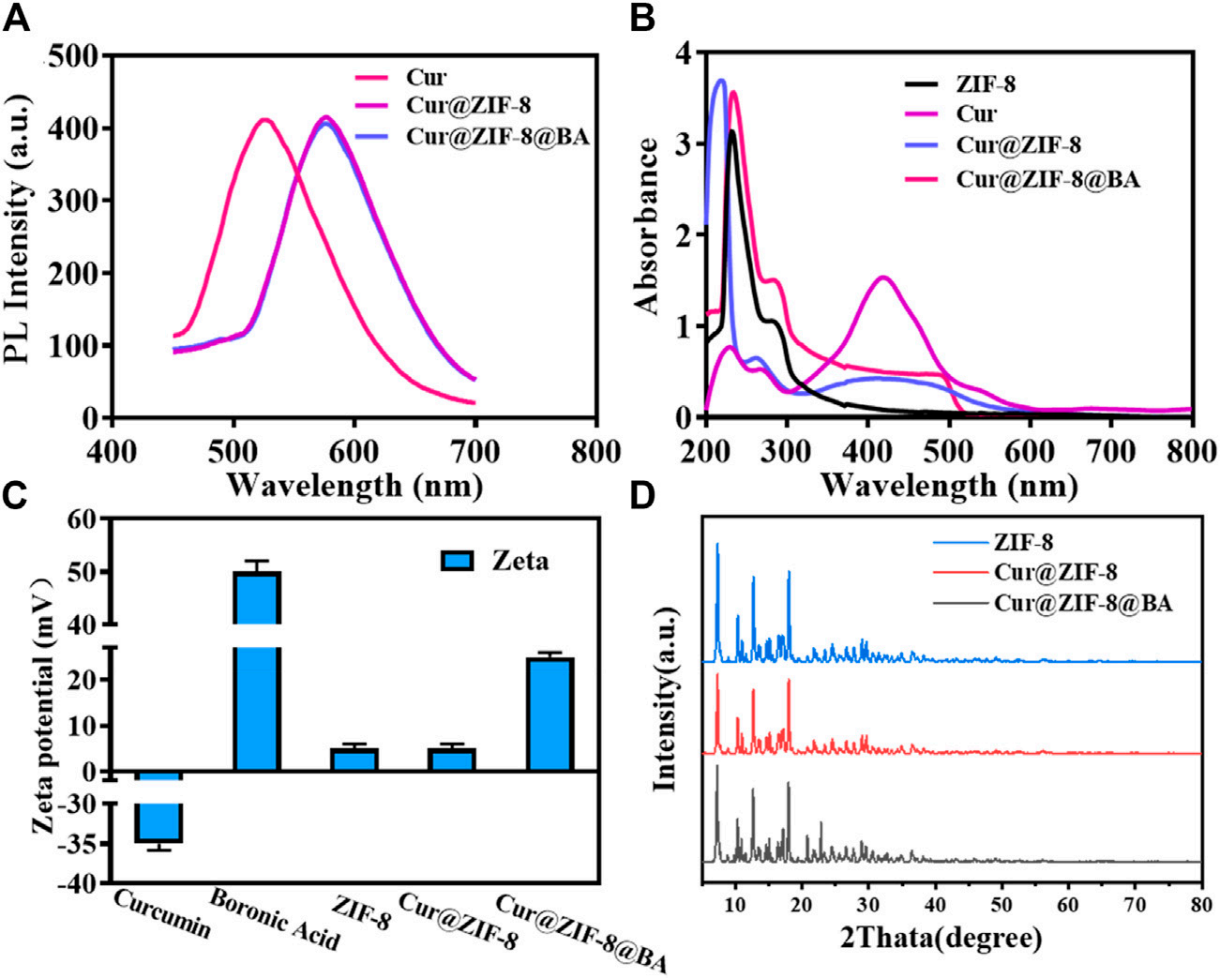
Chemical properties and structural characterization of Cur@ZIF-8@BA. **(A)** PL spectra of Cur, Cur@ZIF-8, and Cur@ZIF-8@BA under the excitation of 420 nm. **(B)** UV-vis absorption spectra of the Cur, ZIF-8, Cur@ZIF-8, and Cur@ZIF-8@BA. **(C)** Zeta potential maps of Cur, BA, ZIF-8, Cur@ZIF-8, and Cur@ZIF-8@BA. **(D)** XRD patterns of simulated ZIF-8, Cur@ZIF-8, and Cur@ZIF-8@BA.

The exceptional optical properties of Cur were investigated in this study by employing fluorescence and UV-VIS spectroscopy to confirm its encapsulation within ZIF-8. The fluorescence spectra demonstrate a robust emission at 545 nm for free Cur, while the emission spectra of Cur@ZIF-8 exhibit a redshift to 625 nm. This shift may be attributed to the reduction in the band gap of the π-π* electron transition of Cur following its interaction with Zn^2+^ in ZIF-8 (Meng et al., 2022). Similarly, the emission spectrum of Cur@ZIF-8@BA also exhibits a peak at 625 nm, indicating that the fluorescence spectrum remains unaffected by BA modification (Figure 1A). The UV-VIS spectra revealed a distinct UV absorption peak at 425 nm for free Cur. However, upon encapsulation within ZIF-8, the ultraviolet light absorption was significantly diminished (Luan et al., 2019; Meng et al., 2022). This can be attributed to the influence of ZIF-8’s pore structure and the presence of Zn^2+^ on the molecular conformation and electron cloud distribution of Cur, resulting in alterations in its UV absorption intensity. Notably, ZIF-8, Cur@ZIF-8, and Cur@ZIF-8@BA exhibited prominent absorption peaks at 228 nm corresponding to the ultraviolet absorption of ZIF-8 (Figure 1B) (Bethi et al., 2023). These optical properties confirm the successful encapsulation of Cur within ZIF-8.

**FIGURE 2:**
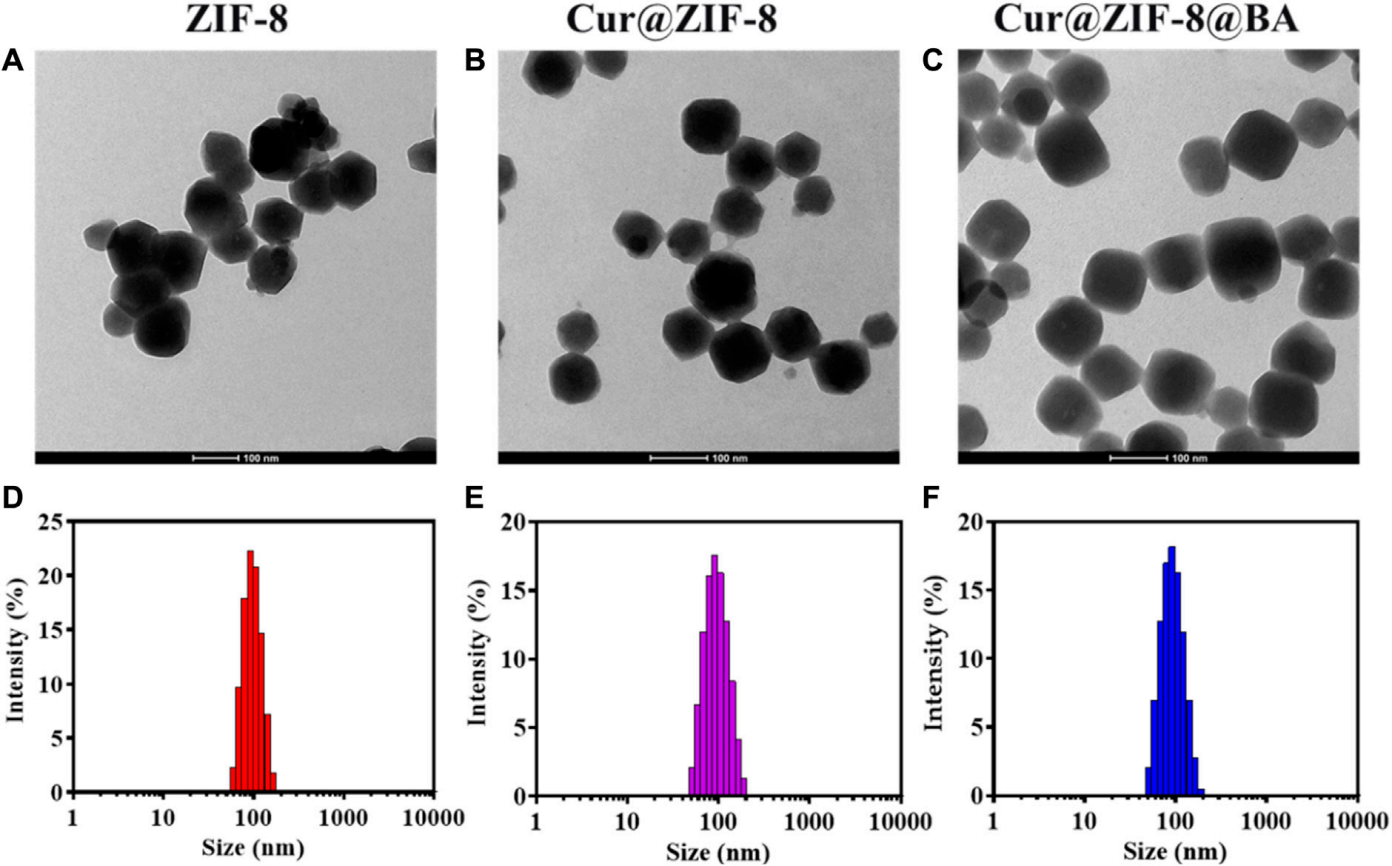
Characterization of Cur@ZIF-8@BA: **(A–C)** Transmission electron microscope images of ZIF-8 **(A)**, Cur@ZIF-8 **(B)**, and Cur@ZIF-8@BA **(C)**, scale bar = 100 nm. **(D–F)** DLS analysis of ZIF-8 **(D)**, Cur@ZIF-8 **(E)**, and Cur@ZIF-8@BA **(F)**.

The Zeta potential results demonstrated that the values for Cur, BA, ZIF-8, Cur@ZIF-8, and Cur@ZIF-8@BA were −35, +50, +5, +5, and +26 mV respectively. Comparable findings have been previously reported (Figure 1C). The XRD analysis results reveal that the characteristic peaks of ZIF-8 are observed at 7.31°, 10.34°, 12.70°, and 17.99°, which aligns with the findings documented in the literature (Xia et al., 2022). Furthermore, Cur@ZIF-8 and Cur@ZIF-8@BA exhibited these identical characteristic peaks, indicating that the incorporation of Cur and BA does not compromise the structural integrity of the ZIF-8 crystal lattice (Figure 1D). This finding underscores the compatibility of our composite material, suggesting that the functionalization with Cur and BA preserves the beneficial properties of ZIF-8 while potentially enhancing its functionality for targeted applications. The results obtained from transmission electron microscopy (TEM) clearly demonstrate the well-defined dodecahedral crystal structure of ZIF-8, which is consistent with previous reports (Zhao et al., 2020). It should be emphasized that the morphology and size of Cur@ZIF-8 remain unaltered compared to ZIF-8, indicating the self-assembly of Cur within the ZIF-8 cavity. Conversely, Cur@ZIF-8@BA exhibits a tendency towards spherical shape and an increase in particle size due to surface modification of ZIF-8 particles by BA (Figures 2A–C). Dynamic light scattering (DLS) analysis further supports these observations as both ZIF-8 and Cur@ZIF-8 predominantly exhibit particle sizes around 105 nm, whereas Cur@ZIF-8@BA primarily shows particle sizes around 122 nm, consistent with TEM results (Figures 2D–F).

The successful synthesis of Cur@ZIF-8@BA was confirmed through optical property detection, XRD analysis, transmission electron microscopy, DLS measurements, and other analytical techniques.

### 3.2 pH dependence and photodynamic performance test of Cur@ZIF-8@BA

The standard curves for free Cur and BA were established by measuring their absorbance at 425 and 280 nm across various concentrations. Results indicated that both Cur and BA exhibited a strong linear relationship with UV absorption within the range of 0.625–10 μg/mL, with R^2^ values of 0.9999 (Supplementary Figures S1A, B) and 0.9985 (Supplementary Figures S1A, B), respectively. These standard curves were utilized to calculate binding efficiency at Cur@ZIF-8@BA, revealing DLC and DLE values of 78.86% and 7.87% for Cur, as well as DLC values of 30.17% and DLE values of 6.56% for BA.

**FIGURE 3:**
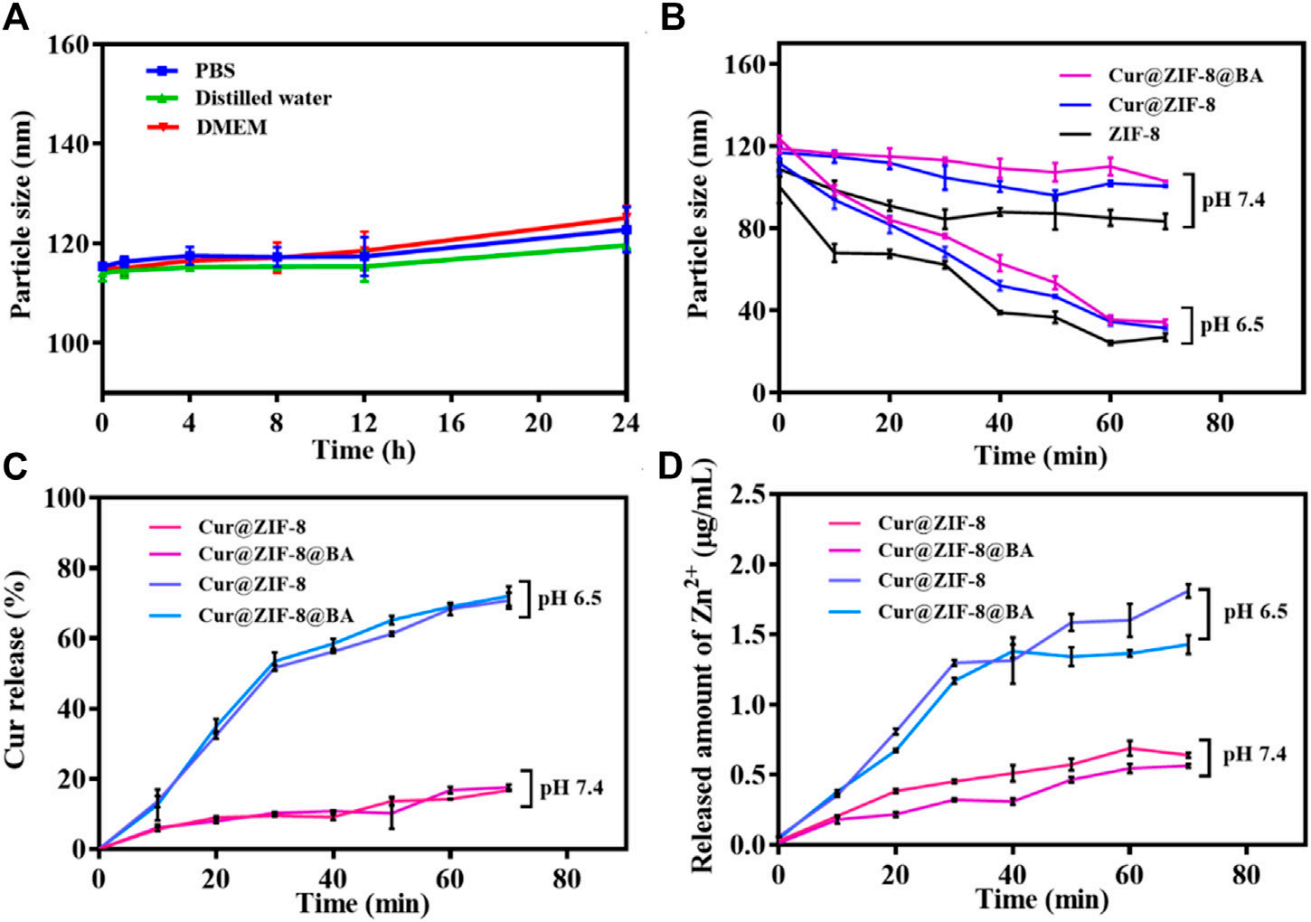
Stability and pH-responsive of Cur@ZIF-8 and Cur@ZIF-8@BA. **(A)** Particle size of Cur@ZIF-8 and Cur@ZIF-8@BA in PBS, distilled water, and DMEM at different times. **(B)** Particle size of ZIF-8, Cur@ZIF-8 and Cur@ZIF-8@BA in PBS solution at pH 6.5 and 7.4 for different times. **(C)** Curcumin release profiles from nanomaterials Cur@ZIF-8 and Cur@ZIF-8@BA in PBS solution (containing 20% ethanol v/v) at pH 6.5 and 7.4. **(D)** Zn^2+^ release from Cur@ZIF-8 and Cur@ZIF-8@BA in PBS solution (pH 6.5 and 7.4).

The essential stability of the material is crucial for its antibacterial properties (He et al., 2015). In this study, the changes in particle size of Cur@ZIF-8@BA was examined following incubation in different media to assess its stability. The findings revealed that Cur@ZIF-8@BA remained intact even after being exposed to PBS, deionized water, and high glucose medium DMEM for 24 h, demonstrating its commendable physiological stability (Figure 3A). Next, the pH response characteristic of Cur@ZIF-8@BA was examined, which is crucial for its ability to decompose and release Zn^2+^ and Cur, thereby exerting a synergistic therapeutic effect in the weakly acidic environment of bacterial infection (Tan et al., 2022). The ZIF-8, Cur@ZIF-8 and Cur@ZIF-8@BA samples were incubated in PBS solutions with varying pH values, and the particle size of the materials was determined over a specific time period. The particle size of these samples exhibited minimal changes upon incubation in PBS at pH = 7.4. In contrast, a significant reduction in their particle size was observed with increasing incubation time at pH = 6.5 (Figure 3B). The findings suggest that the material has the ability to decompose and exhibit antibacterial activity in a weakly acidic environment, which is consistent with previous reports (Tan et al., 2022). The pH-dependent disintegration of ZIF-8 leads to the release of its contents. Therefore, we assessed the release of Cur and Zn^2+^ from the material under different pH conditions. The results demonstrated that both Cur and Zn^2+^ release increased with longer incubation time at pH 6.5 for Cur@ZIF-8 and Cur@ZIF-8@BA, indicating a time-dependent disintegration of the materials. In contrast, only minimal amounts of Cur and Zn^2+^ leaching were observed during the test period at pH = 7.4 (Figures 3C, D). In addition to Cur and Zn^2+^, the presence of BA in Cur@ZIF-8@BA enhances its bacterial binding affinity. This highlights the unique pH-responsive release properties and ability of Cur@ZIF-8@BA to target the bacterial microenvironment.

**FIGURE 4:**
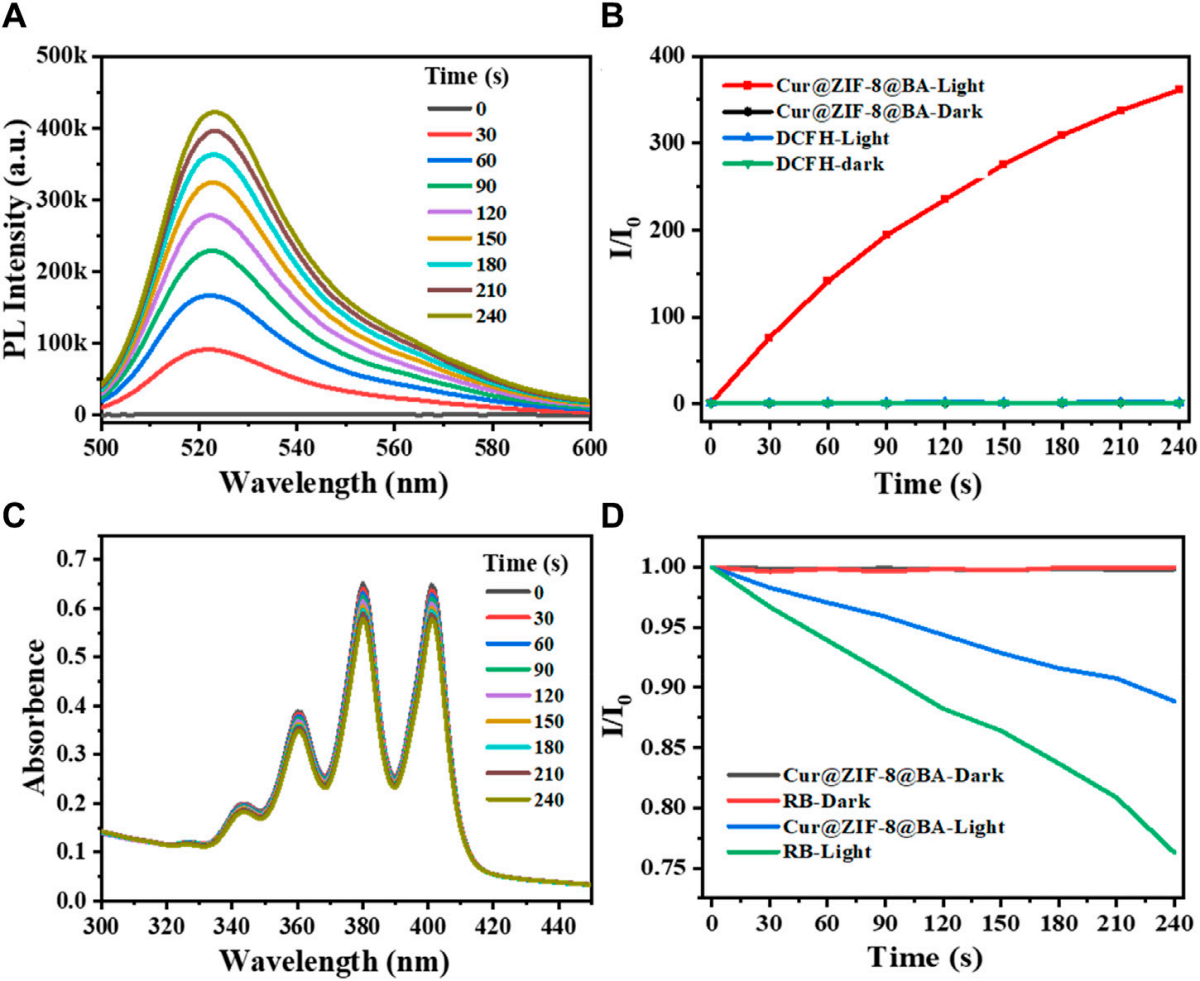
Chemical trapping of the ROS and ^1^O_2_ generation. **(A)** Photoactivation of DCFH with Cur@ZIF-8@BA. The measurements were carried out under white light irradiation in PBS buffer. **(B)** Activation rates of DCFH with light irradiation under different conditions at 525 nm fluorescence emission. **(C)** Photodegradation of ABDA with Cur@ZIF-8@BA. The measurements were carried out under white light irradiation in PBS buffer. **(D)** Decomposition rates of ABDA with light irradiation under different conditions at 378 nm absorbance.

The strategy of antimicrobial photodynamic therapy involves the utilization of photosensitizers to generate a substantial quantity of reactive oxygen species (ROS) upon exposure to light, thereby effectively eradicating bacteria (Xiao et al., 2022). The commercial probes DCFH-DA and ABDA were employed for the detection of ROS and singlet oxygen (^1^O_2_) generation, respectively, following treatment with Cur@ZIF-8@BA (Liu et al., 2022; Xu et al., 2022). The results demonstrated that the fluorescence intensity of the Cur@ZIF-8@BA-treated solution increased in correlation with illumination time, indicating a light-dependent ROS generation. Conversely, no significant change in fluorescence intensity was observed for DCFH-DA and unilluminated Cur@ZIF-8@BA (Figures 4A, B). Consistent with this observation, there was a notable alteration in UV absorption spectra of solutions treated with Cur@ZIF-8@BA over time, revealing a decrease in absorption intensity which further confirmed light-dependent singlet oxygen generation (Rose Bengal served as a control). Unilluminated Cur@ZIF-8@BA and Rose Bengal did not generate any ^1^O_2_ (Figures 4C, D). The results suggest that Cur, functioning as a natural photosensitizer, exhibits excellent performance in generating reactive oxygen species (ROS), and its ROS production remains unaffected by the encapsulation of ZIF-8 and the modification of BA.

### 3.3 Evaluation of antibacterial activity of Cur@ZIF-8@BA *in vitro*

**FIGURE 5:**
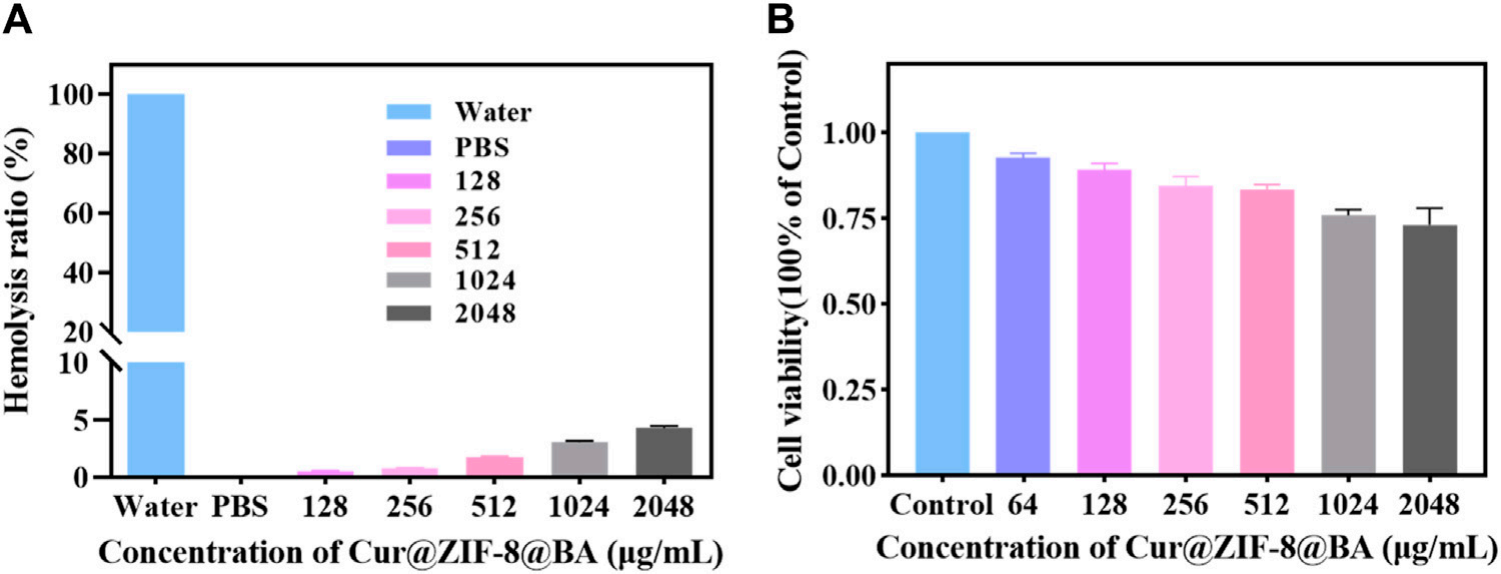
**(A)** Effect of Cur@ZIF-8@BA at various concentrations on the hemolysis rate. **(B)** Cytotoxicity of Cur@ZIF-8@BA at various concentrations in NIH 3T3 cells for 24 h.

Before conducting antimicrobial activity testing, the material is initially evaluated for safety (Liu et al., 2016). Firstly, the cytotoxicity of Cur@ZIF-8@BA was assessed using Cell Counting Kit-8. The results demonstrated that Cur@ZIF-8@BA exhibited negligible toxicity towards NIH 3T3 cells, with a cell viability of 73.25% even at a high concentration of 2,048 μg/mL (Figure 5A). The blood compatibility of the material was subsequently investigated to assess the potential risks associated with Cur@ZIF-8@BA entering the systemic circulation and coming into contact with blood during treatment. The hemolysis test results demonstrated that even at high concentrations (2,048 μg/mL), the hemolysis rate of Cur@ZIF-8@BA was merely 4.32%, which falls within the acceptable safety range (Figure 5B) (Zhou et al., 2022).

**FIGURE 6:**
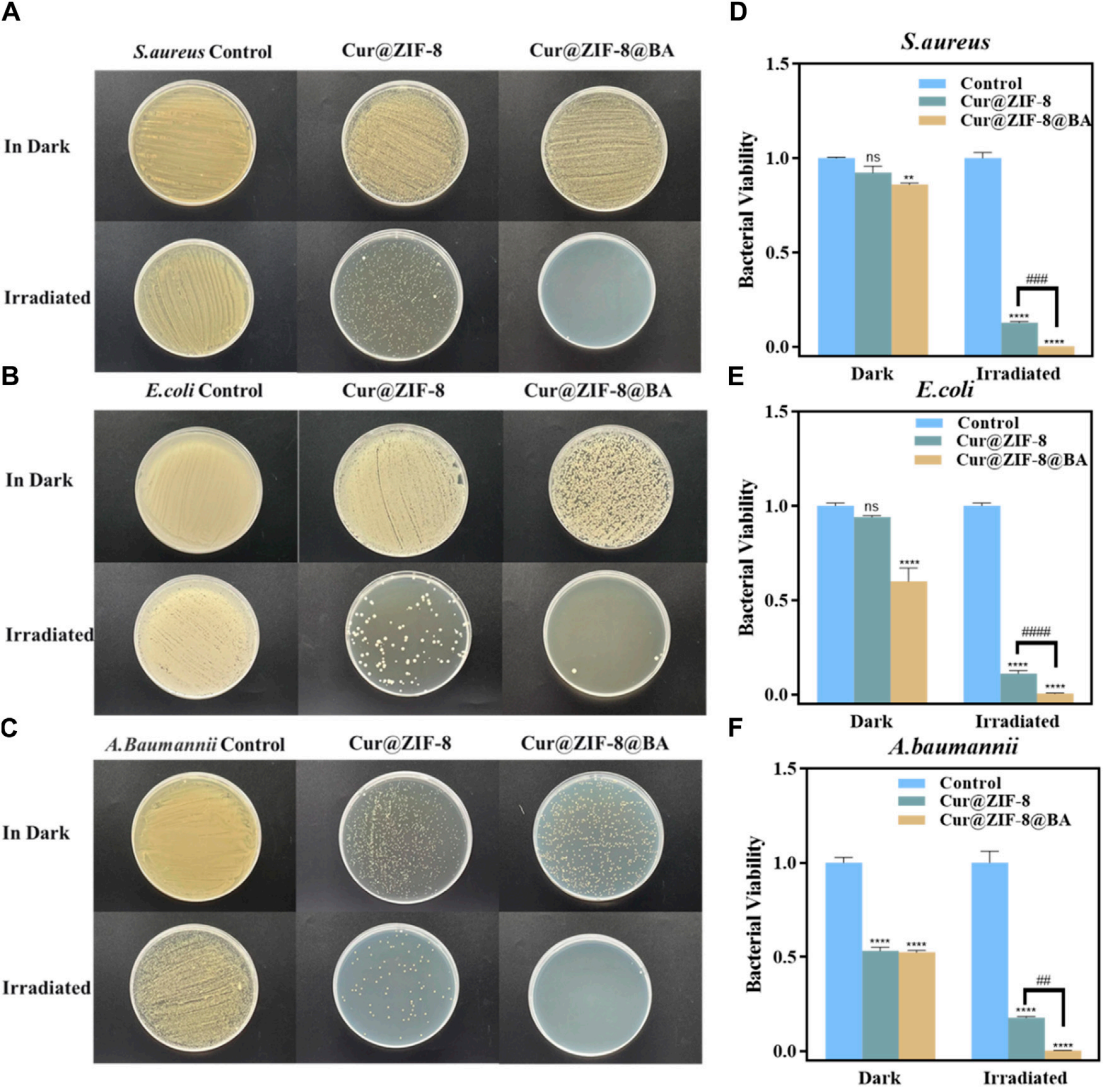
*In vitro* antimicrobial performance experiments of nanomaterials Cur@ZIF-8 and Cur@ZIF-8@BA. Plates of **(A)** *S. aureus* **(B)** *E. coli* and **(C)** *A. baumannii* treated with 64 μg/mL Cur@ZIF-8 and Cur@ZIF-8@BA under 100 mW/cm^2^ white light for 1 h and dark conditions. Survival of **(D)** *S. aureus* **(E)** *E. coli* and **(F)** *A. baumannii* treated with 64 μg/mL Cur@ZIF-8 and Cur@ZIF-8@BA under white light and dark conditions. ^ns^ *p* > 0.05, **p < 0.01, ****p < 0.0001, vs. Control group; ^##^ *p* < 0.01, ^###^ *p* < 0.001 and ^####^ *p* < 0.0001.

**FIGURE 7:**
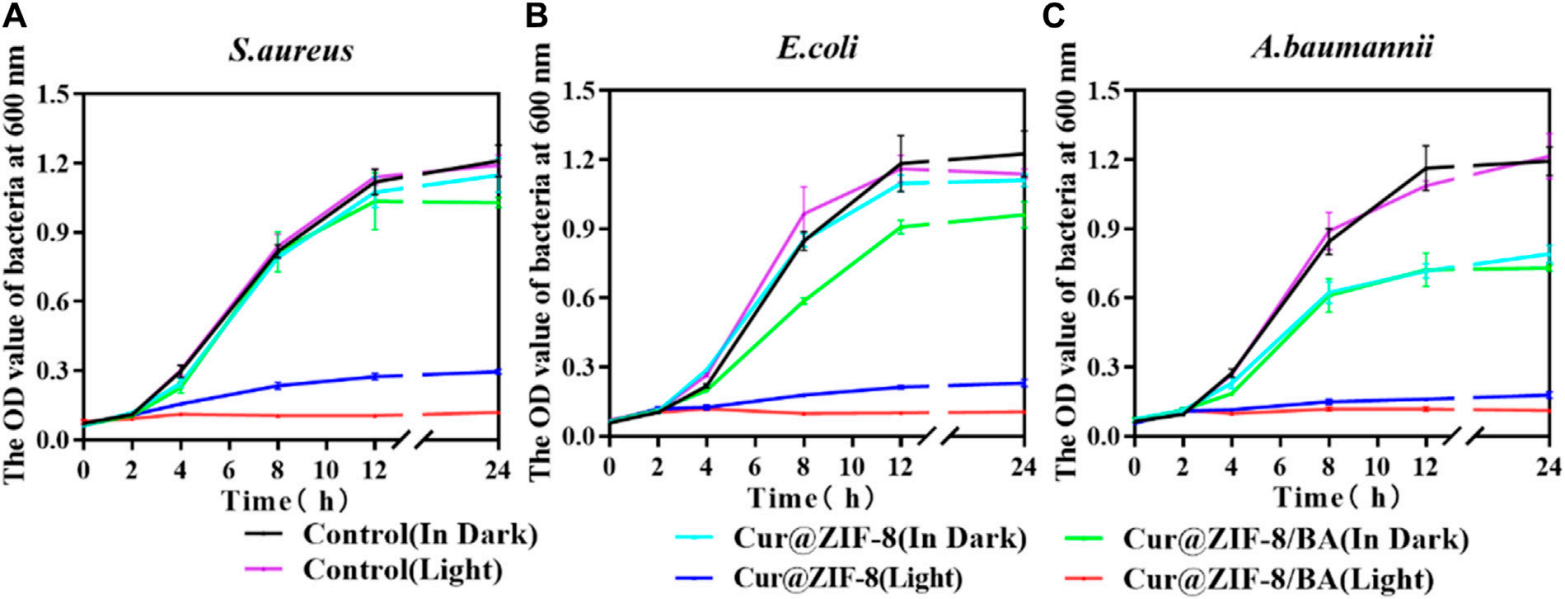
Growth curves of 64 μg/mL Cur@ZIF-8 and Cur@ZIF-8@BA-treated *S. aureus* **(A)**, *E. coli* **(B)** and *A. baumannii* **(C)** in light irradiation and dark.

On the basis of demonstrating the excellent safety profile of Cur@ZIF-8@BA, we proceeded to determine the minimum inhibitory concentration (MIC) values of Cur@ZIF-8 and Cur@ZIF-8@BA in order to evaluate their antibacterial activities. The results demonstrated that compared to a dark environment, the MIC values of Cur@ZIF-8@BA against *S. aureus*, *E. coli* and *A. baumannii* decreased by 4-fold, 8-fold, and 16-fold respectively. Similarly, the MIC values of Cur@ZIF-8 against these bacteria decreased by 2-fold, 4-fold, and 8-fold respectively. This phenomenon can be attributed to the abundant production of reactive oxygen species (ROS) by Cur under light conditions which effectively sterilizes bacteria (Seidi Damyeh et al., 2020). Additionally, the modification of BA enhances the bacterial affinity of Cur@ZIF-8@BA, thereby conferring stronger antibacterial properties compared to Cur@ZIF-8 (Table 1) (Guo et al., 2022). The antibacterial activity of the material was further confirmed through an AGAR plate experiment. The results demonstrated a significantly higher inhibition of *S. aureus*, *E. coli* and *A. baumannii* in the light group compared to the non-light group. Specifically, Cur@ZIF-8 exhibited inhibition rates of 86.84%, 88.36% and 92.63% against *S. aureus*, *E. coli* and *A. baumannii* in light group respectively. Furthermore, Cur@ZIF-8@BA displayed even higher inhibition rates of 99.99%, 98.72% and 99.75% against these three bacteria strains respectively, indicating that the modification with BA can enhance the antibacterial properties of the materials a novel finding in our study (Figures 6A–F). This observation was further supported by the growth curve analysis which revealed that Cur@ZIF-8@BA exerted superior inhibitory effects on bacterial growth among all tested strains (Figures 7A–C). It is noteworthy that Cur@ZIF-8 and Cur@ZIF-8@BA exhibited certain antibacterial activity even in the absence of light, as observed in the MIC, AGAR plate, and growth curve experiments. We postulate that this may be attributed to the disintegration of Cur@ZIF-8 and Cur@ZIF-8@BA, leading to the release of Zn^2+^ and BA for bacterial killing. Conversely, under light conditions, Cur is activated to generate ROS which synergistically interacts with Zn^2+^ and BA for optimal antibacterial efficacy.

**TABLE 1:** The minimum inhibitory concentration (MIC) of Cur@ZIF-8 and Cur@ZIF-8@BA against *S. aureus*, *E. coli* and *A. baumannii* under dark or light irradiation.

Strains	Cur@ZIF-8 (dark)	Cur@ZIF-8@BA (dark)	Cur@ZIF-8 (irradiated)	Cur@ZIF-8@BA (irradiated)
*E. coli*	512 μg/mL	256 μg/mL	128 μg/mL	64 μg/mL
*S. aureus*	256 μg/mL	256 μg/mL	128 μg/mL	64 μg/mL
*A. baumannii*	512 μg/mL	256 μg/mL	64 μg/mL	32 μg/mL

### 3.4 Study on antibacterial mechanism of Cur@ZIF-8@BA

**FIGURE 8:**
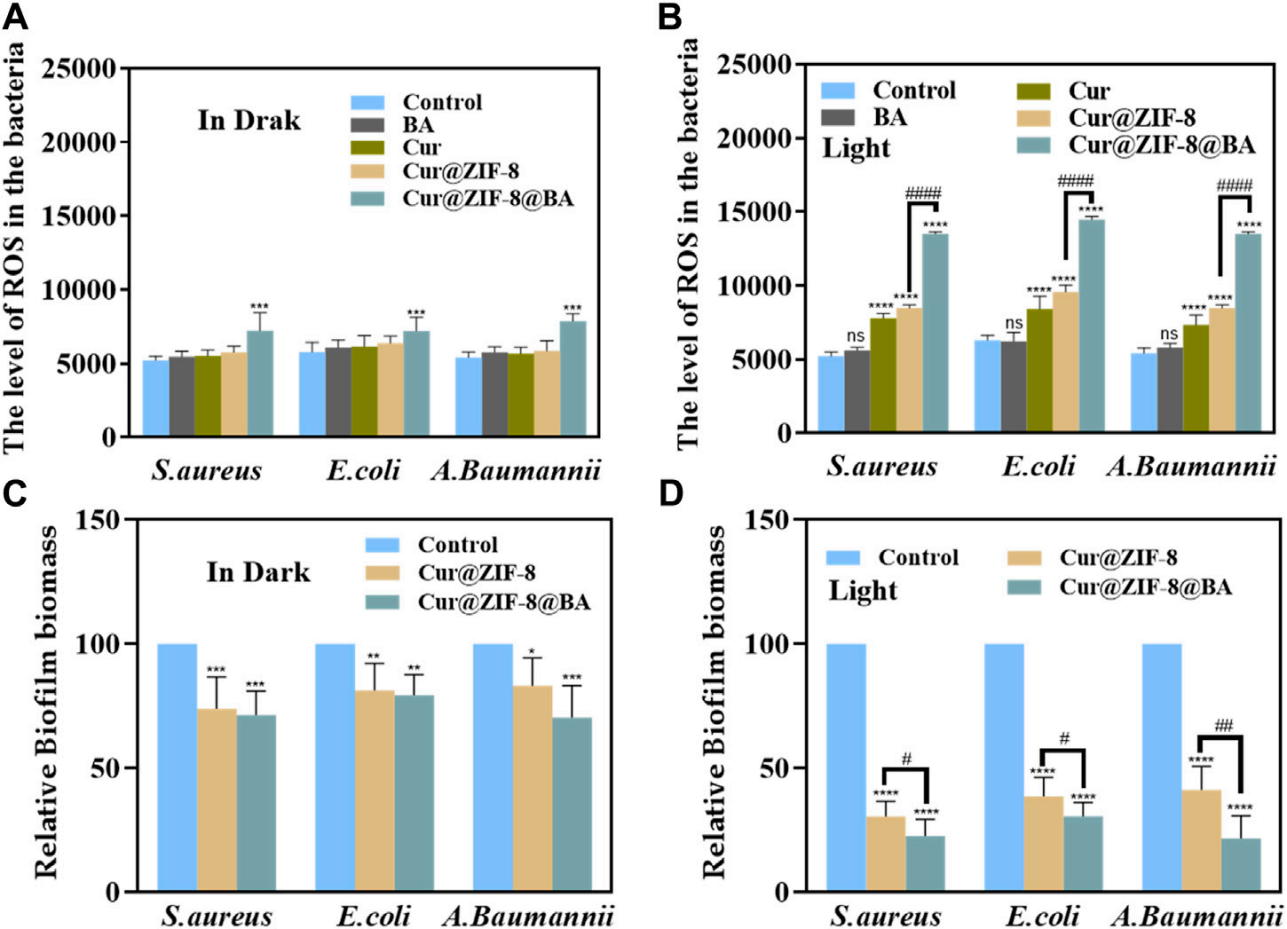
The ROS levels in *S. aureus*, *E. coli* and *A. baumannii* after incubation with 64 μg/mL BA, Cur, Cur@ZIF-8 and Cur@ZIF-8@BA for 1 h under dark **(A)** and light irradiation **(B)**. Effectiveness of nanomaterials Cur@ZIF-8 and Cur@ZIF-8@BA (64 μg/mL) in removing biofilms of *S. aureus*, *E. coli* and *A. baumannii* under dark **(C)** or light irradiation **(D)**. ^ns^ *p* > 0.05, *p < 0.05, **p < 0.01, ***p < 0.001, and ****p < 0.0001, vs. Control group; ^#^ *p* < 0.05, ^##^ *p* < 0.01, ^###^ *p* < 0.001 and ^####^ *p* < 0.0001.

The ROS levels were determined by incubating bacteria with Cur, BA, Cur@ZIF-8 and Cur@ZIF-8@BA, followed by light/dark treatment, in order to further validate the ability of Cur@ZIF-8@BA to stimulate photosensitizer Cur for ROS production, leading to effective inactivation of pathogenic microorganisms through destruction of their cell membrane, proteins, DNA, and other biological macromolecules (Ni et al., 2022). The results demonstrated that, in the absence of light, only Cur@ZIF-8@BA exhibited a significant difference in ROS levels between the Control group, which can be attributed to the synergistic effect of Zn^2+^, BA, and other components. Conversely, upon illumination, ROS levels were significantly higher in Cur, Cur@ZIF-8, and Cur@ZIF-8@BA compared to the Control group. Additionally, ROS levels in Cur@ZIF-8@BA were also notably higher than those in the Cur@ZIF-8 group. This outcome highlights how BA enhances bacterial binding affinity while improving Cur photodynamic therapy (Figures 8A, B). The aforementioned findings once again demonstrate the ability of Cur to generate an ample amount of reactive oxygen species, thereby synergistically combating bacteria in a complex infectious milieu with the assistance of Zn^2+^ and BA.

One challenge in the treatment of bacterial infections lies in the impediment posed by bacterial biofilms to drug penetration, thereby significantly compromising the bactericidal efficacy (Hu et al., 2021). Consequently, eliminating biofilms can enhance the sterilizing activity of drugs. Photodynamic therapy (PDT) is regarded as a promising approach for inhibiting or eradicating biofilms (Orlandi et al., 2021). In this study, we investigated the capacity of Cur@ZIF-8 and Cur@ZIF-8@BA to clear biofilms. The results demonstrated that, under light conditions, the application of Cur@ZIF-8 and Cur@ZIF-8@BA significantly reduced the level of bacterial biofilm compared to the Control group. This reduction was attributed to the generation of a substantial amount of reactive oxygen species (ROS) by Cur during photodynamic therapy. Moreover, it was observed that Cur@ZIF-8@BA exhibited a slightly superior therapeutic effect compared to Cur@ZIF-8, possibly due to enhanced binding with bacteria facilitated by BA modification. Additionally, BA itself possessed certain permeability properties and could disrupt bacterial biofilms (Abid et al., 2024). Similarly, even under dark conditions, both Cur@ZIF-8 and Cur@ZIF-8@BA displayed some degree of biofilm clearance which could be attributed to the permeability properties of BA and Zn^2+^ to biofilms (Figures 8C, D).

## 4 Conclusion

We have developed a nano-antibacterial agent, Cur@ZIF-8@BA, with pH-responsive and photodynamic therapy, which enhances the binding affinity between the material and bacteria, significantly augments the Cur-mediated antibacterial activity. Moreover, Cur@ZIF-8@BA exhibits long-term physiological stability and low toxicity, thereby demonstrating its potential for further advancement. Collectively, our study presents a promising avenue for utilizing photodynamic therapy in the treatment of bacterial infections.

## Data Availability

The raw data supporting the conclusions of this article will be made available by the authors, without undue reservation.
