# Supplementary material for: Cur@ZIF-8@BA nanomaterials with pH-responsive and photodynamic therapy properties promotes antimicrobial activity
Source: Front Chem. 2024 Jun 24;12:1417715. doi: 10.3389/fchem.2024.1417715 (PMC11228171; doi:10.3389/fchem.2024.1417715)
Supplement: Supplementary file 1 [file DataSheet1.docx]

Supplementary Material


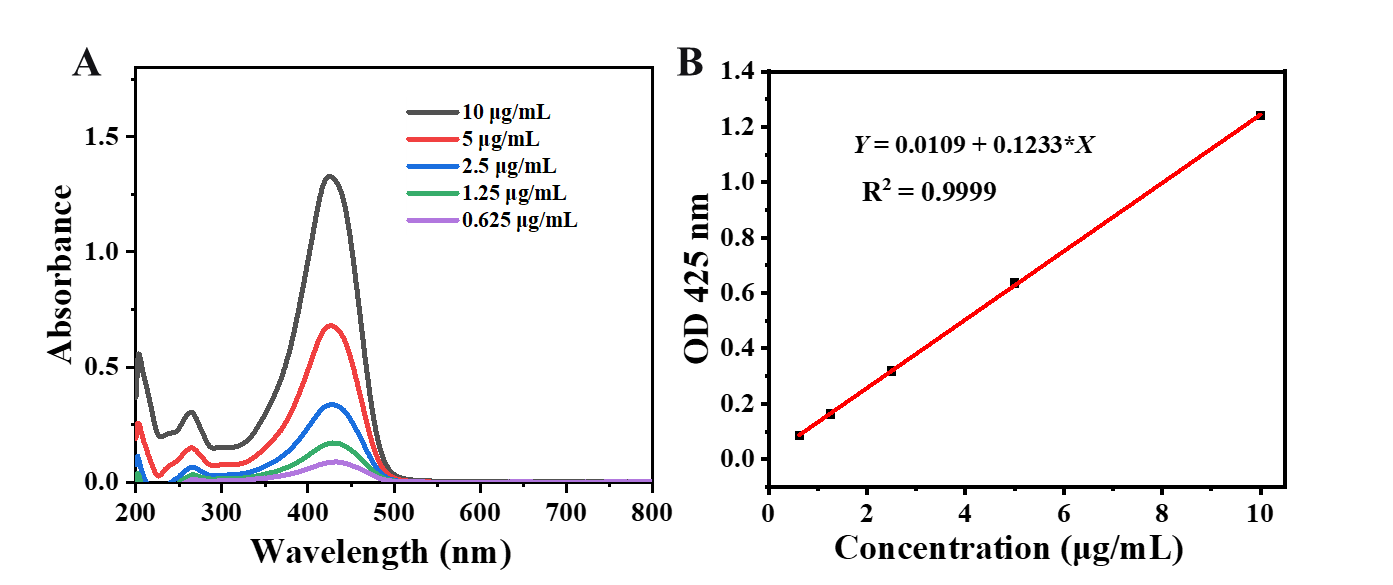


**FIG. S1 (A)** Ultraviolet absorption spectra of free curcumin with different concentrations (0.625−10.0 μg/mL); **(B)** The linear relationship between the concentration of Cur in ethanol and absorption intensity.


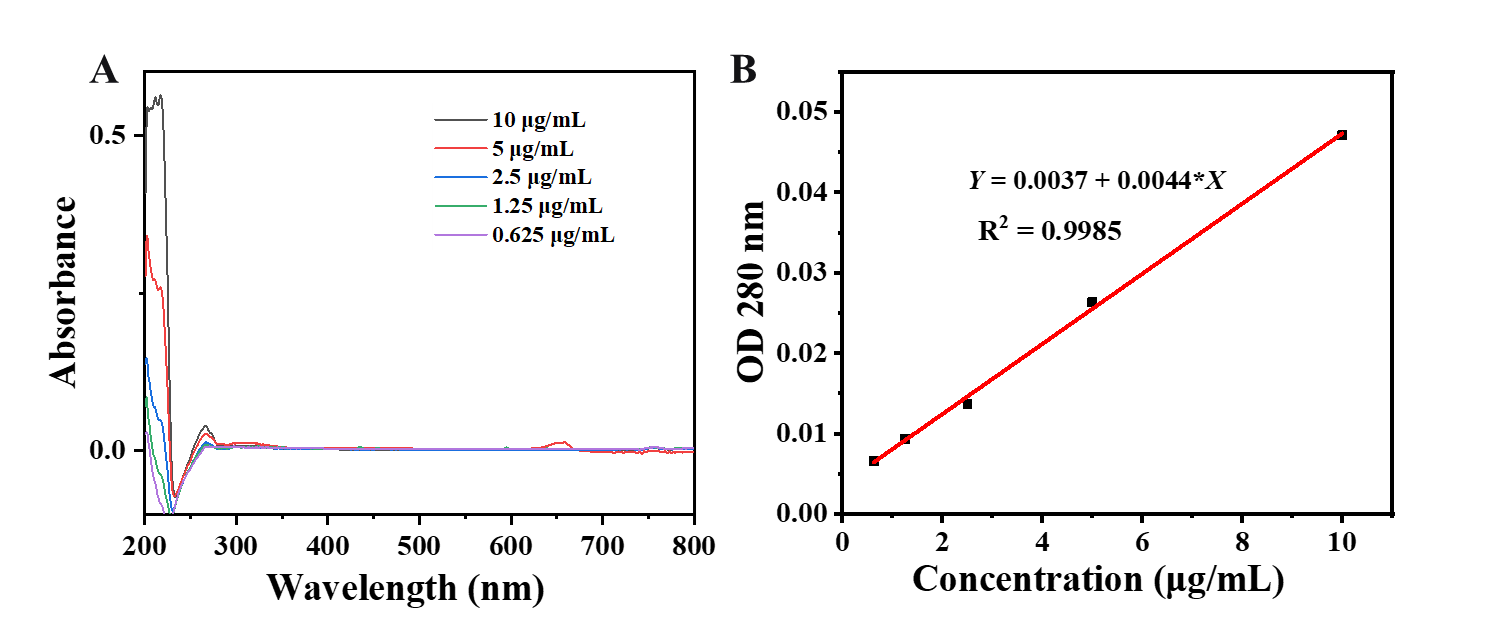


**FIG. S2 (A)** Ultraviolet absorption spectra of free BA with different concentrations (0.625−10.0 μg/mL); **(B)** The linear relationship between the concentration of BA in ethanol and absorption intensity.
